# Integrative prognostic modeling for stage III lung adenosquamous carcinoma post-tumor resection: machine learning insights and web-based implementation

**DOI:** 10.3389/fsurg.2024.1489040

**Published:** 2024-10-22

**Authors:** Min Liang, Peimiao Li, Shangyu Xie, Xiaoying Huang, Xiaocai Li, Shifan Tan

**Affiliations:** ^1^ Department of Respiratory and Critical Care Medicine, Maoming People’s Hospital, Maoming, China; ^2^ Center of Respiratory Research, Maoming People’s Hospital, Maoming, China; ^3^ Department of General Internal Medicine, Kangmei Hospital, Puning, China

**Keywords:** machine learning, prognosis, survival, adenosquamous carcinoma, primary tumor, resection

## Abstract

**Introduction:**

The prognostic landscape of stage III Lung Adenosquamous Carcinoma (ASC) following primary tumor resection remains underexplored. A thoughtfully developed prognostic model has the potential to guide clinicians in patient counseling and the formulation of effective therapeutic strategies.

**Methods:**

Utilizing data from the Surveillance, Epidemiology, and End Results database spanning 2000 to 2018, this study identified independent prognostic factors influencing Overall Survival (OS) in ASC using Boruta analysis. Employing Gradient Boosting, Random Forest, and Neural Network algorithms, predictive models were constructed. Model performance was assessed through key metrics, including Area Under the Receiver Operating Characteristic Curve (AUC), calibration plot, Brier score, and Decision Curve Analysis (DCA).

**Results:**

Among 241 eligible patients, seven clinical parameters—age, sex, primary tumor size, N stage, primary tumor site, chemotherapy, and systemic therapy—were identified as significant predictors of OS. Advanced age, male gender, larger tumor size, absence of chemotherapy, and lack of systemic therapy were associated with poorer survival. The Random Forest model outperformed others, achieving 3- and 5-year AUCs of 0.80/0.79 (training) and 0.74/0.65 (validation). It also demonstrated better calibration, lower Brier scores (training: 0.189/0.171; validation: 0.207/0.199), and more favorable DCA. SHAP values enhanced model interpretability by highlighting the impact of each parameter on survival predictions. To facilitate clinical application, the Random Forest model was deployed on a web-based server for accessible prognostic assessments.

**Conclusions:**

This study presents a robust machine learning model and a web-based tool that assist healthcare practitioners in personalized clinical decision-making and treatment optimization for ASC patients following primary tumor resection.

## Introduction

Globally, lung cancer persists as a substantial public health challenge. According to the Global Burden of Disease Study report, the year 2019 witnessed an incidence of over 2.26 million new cases and 2.04 million deaths attributed to lung cancer (1, 2). Primary lung adenosquamous carcinoma (ASC) constitutes a rare subtype, representing approximately 0.4%–4% of all lung carcinoma (3, 4). The 2015 World Health Organization (WHO) classification of lung tumors characterizes ASC as “a carcinoma demonstrating features of both squamous cell carcinoma (SCC) and adenocarcinoma (ADC), with each component constituting at least 10% of the tumor” (5). Notwithstanding its composite nature incorporating both ADC and SCC elements, ASC exhibits a more aggressive histological comportment, and the reported overall cumulative 5-year survival rate is only 6.2% (6, 7). Therefore, early diagnosis and timely medical intervention are crucial for improving outcomes in ASC.

In accordance with the National Comprehensive Cancer Network Guidelines (Version 1.2020, 2019), individuals identified as suitable candidates for intervention at stages I to II of non-small cell lung cancer (NSCLC) may receive recommendations for surgical intervention, positioned as an optimal therapeutic modality with curative potential in the context of NSCLC (8). However, the lack of a clear recommendation regarding the suitability of surgical treatment for patients with stage III NSCLC, especially those classified as IIIA-N2, creates a significant gap in clinical guidance. Additionally, due to the unique and uncommon nature of ASC, the available research is limited, restricting our understanding of the impact of surgical procedures on the prognosis of stage III patients affected by this subtype. Therefore, a thorough examination of the clinical characteristics and prognostic factors specific to stage III ASC in patients who have undergone tumor resection is crucial. Such an investigation is essential for optimizing the clinical management strategies for this condition.

In recent years, there has been a growing emphasis on applying advanced computational technologies such as artificial intelligence (AI) (9), the Internet of Things (IoT) (10), and deep learning algorithms in medicine (11). Machine learning methodologies, renowned for their ability to autonomously extract insights from extensive datasets, facilitate the identification of subtle relationships between variables and clinical outcomes. This unique capability allows for the creation of highly effective models proficient in predicting outcomes for previously unexplored datasets (12). However, despite these advancements, there is a noticeable gap in the current body of knowledge concerning the development of machine learning-based models tailored specifically to identify independent prognostic features in surgical ASC patients. Similarly, the application of machine learning methodologies to predict survival probabilities within this specific patient cohort has not received adequate attention in existing research.

In this study, we utilized data from the SEER database and various machine learning techniques to develop prognostic models for evaluating survival probability in stage III ASC patients undergoing primary tumor resection. Additionally, we conducted a comprehensive comparative analysis to evaluate the effectiveness of these machine learning models compared to the concurrently used TNM staging and Cox regression systems. The overarching objective was to identify the model that demonstrated superior performance in prognostication. As a result, this investigation culminated in the development of a web-based classifier enriched with visual representations, enhancing both accessibility and utility in the clinical context.

We acknowledge that the rarity of ASC and the retrospective nature of SEER data present challenges, such as limited sample sizes and potential biases inherent in registry data. Despite these limitations, our study provides valuable insights and a foundation for future research to improve prognostication and management of stage III ASC patients.

## Methods

### Compliance with ethics guidelines

This article is dependent upon open-access databases and does not involve original research incorporating human participants or animals. To safeguard patient privacy and uphold ethical principles, each author has formally affirmed the adherence to the Surveillance, Epidemiology, and End Results (SEER) research data agreement.

### Data extraction

The present retrospective cohort study employs data extracted from the Surveillance, Epidemiology, and End Results (SEER) database. Administered by the National Cancer Institute (NCI), this database comprises 18 population-based cancer registries, encompassing almost 28% of the U.S. population. Capitalizing on the extensive coverage afforded by the SEER program, our study derives advantage from an inclusive depiction of clinicopathological details, tumor characteristics, and therapeutic interventions. The clinical data of patients were acquired through the utilization of the SEER Stat software (version 8.4.0.1; https://seer.cancer.gov/data-software/) to ensure the precision and reliability of the information obtained.

### Study population

The inclusion criteria for this study were as follows: (i) individuals who received a pathologically validated diagnosis of ASC and underwent primary tumor resection from 2000 to 2018. ASC was defined in accordance with the third edition of the International Classification of Diseases for Oncology (ICD-O-3), specifically identified by site codes C34.0-C34.9 and histological type code 8560/3; (ii) patients with a solitary primary tumor and no concurrent primary tumors in other anatomical sites; The exclusion criteria encompassed the following: (i) inadequate demographic particulars, involving incomplete data on age, gender, ethnicity, and marital status; (ii) insufficient clinicopathological details, including histologic classification, precise measurement of primary tumor dimensions, primary tumor location, tumor laterality, histological differentiation grade, as well as TNM stage; (iii) incomplete therapeutic information concerning surgical methods for the primary tumor (such as wedge, lobectomy, bilobectomy, and pneumonectomy), surgery of other regions, regional nodes examination, systemic therapy during the perioperative period, and chemotherapy or radiotherapy during the disease course; and (iv) lack of data regarding survival status and follow-up.

### Baseline characteristics presentation

This study primarily focused on OS, meticulously defined as the temporal interval from the date of cancer diagnosis to the subsequent date of death, irrespective of the cause. Clinical and demographic features at baseline were systematically presented, with continuous variables summarized using mean and standard deviation, and categorical variables delineated through frequencies and percentages. A total of 18 variables were incorporated in this study to discern independent prognostic features among patients afflicted with ASC. The demographic parameters included age, gender, ethnicity, and marital status. The clinicopathological attributes of the tumor comprised tumor laterality, primary tumor location, tumor size, tumor grade, T stage, N stage, and treatment-related information, including details on surgery, chemotherapy, and radiotherapy.

### Segmentation of study cohort and organization of data structure

To construct and validate the model, eligible patients underwent systematic allocation into two cohorts, namely the training and validation cohorts. This allocation, achieved at an 8:2 ratio using computer-generated random numbers, laid the foundation for subsequent analyses. The data from the training cohort played a pivotal role in crafting a prognostic model and a risk assessment classification system, thus contributing significantly to the robust development of the analytical framework. In contrast, the validation cohort, distinct from the training cohort, played a crucial role in evaluating and validating the model's performance, ensuring the reliability of the study outcomes. In the realm of machine learning, optimization of the classification threshold was carried out through a 5-fold cross-validation procedure applied to the training cohort. The overarching objective was to maximize the Area Under the Receiver Operating Characteristic Curve (AUC), enhancing the model's predictive accuracy and generalizability.

### Feature engineering and model construction strategy

In our analysis, we employed the Boruta model, a wrapper algorithm specifically designed for survival analysis tasks, to assess determinants of survival. The Boruta model distinguishes and selects relevant features by comparing their importance to that of shadow attributes—randomized or permuted versions of the original variables. The retention of features deemed more important than their shadows enhances the robustness of prognostic models by eliminating irrelevant or redundant variables. This contributes to improved interpretability and predictive performance in survival prognosis. Following this, we developed conventional prognostic models using Cox regression methodology, incorporating the survival determinants identified by the Boruta model. Simultaneously, the TNM staging model employed its own determinants for development. To assess the optimal prognostic framework, we utilized the mlr3 package in the R programming environment to train Random Forest, Gradient Boosting Machine, and Neural Network for survival analysis. All models were trained using the same set of survival determinants, and the objective was to determine the most effective prognostic model for 3- and 5-year OS rates among these algorithms.

The study employed the Survival-Synthetic Minority Over-sampling Technique (SMOTE) algorithm, implemented through the “smotefamily” package, to balance the dataset before model training. This algorithm, specifically designed for survival analysis, addresses class imbalances by generating synthetic data to bolster minority event cases. By interpolating new samples between existing event observations, it enhances model sensitivity and accuracy, which is particularly beneficial in fields like medicine and engineering, where accurate prediction of time-to-event outcomes is critical.

Random Forest constructs predictive models by aggregating the outputs of multiple decision trees. We optimized the model using grid search for hyperparameter tuning, adjusting the number of trees (200–500), minimum samples required to split an internal node (15–21), and the number of features considered at each split (mtry, 3–5), tailoring the algorithm to our dataset.

Gradient Boosting builds models by sequentially combining weak learners to minimize prediction errors. We fine-tuned hyperparameters including the number of trees (n.trees, 100–500), maximum interaction depth (1–5), minimum observations in a terminal node (5–21), and the shrinkage parameter (0.001–0.1). The model utilized the Kaplan-Meier estimator and modeled proportional hazards, suitable for survival analysis.

Neural Networks model complex non-linear relationships within data. We configured the network with the Adam optimizer, early stopping with a patience of 20 epochs to prevent overfitting, a batch size of 32, and a maximum of 500 training epochs. Hyperparameters optimized included the number of layers, nodes per layer, learning rate, dropout rate, and weight decay. The trafo function transformed the search space by combining the number of layers and nodes into a composite parameter (num nodes), streamlining the hyperparameter optimization process.

### Model performance evaluation

The model's performance was comprehensively assessed using various evaluation metrics, including Receiver Operating Characteristic (ROC) curve analysis, calibration curve analysis, Decision Curve Analysis (DCA), and the Brier score. The ROC curve, measuring the discriminatory capacity through the Area Under the Curve (AUC), serves as an indicator of precision in prognostication. Calibration analysis evaluates the alignment between predicted and actual probabilities, with an optimal model demonstrating close alignment with the 45° diagonal line in the calibration plot. Decision Curve Analysis was employed to assess clinical efficacy, calculating net benefits at different threshold probabilities. The Brier score, computed by comparing predicted and actual outcomes, represents the mean squared difference and is crucial in evaluating predictive accuracy in survival analysis. The formula for the Brier score in survival analysis, expressed as:

$$\mathrm{Brier}\;\mathrm{Score}\;=\;(1/N)\;*\;\Sigma (\mathrm{Pi}-\mathrm{Oi}{)}^{2}$$

indicates that a lower score corresponds to better predictive accuracy, with zero denoting perfect predictions. This comprehensive evaluation framework ensures a nuanced understanding of the model's prognostic performance across various dimensions.

### Model interpretation

The SHAP (SHapley Additive exPlanations) package played a pivotal role in unraveling the intricacies of machine learning models. In particular, we employed the beeswarm summary plot within the SHAP framework to visually represent the contribution of variables to the model outcomes. Rooted in game-theoretic principles, SHAP serves as a methodological tool designed to provide insights into the outcomes produced by machine learning models. This approach facilitates the identification of predominant features that significantly influence the model's predictions, offering a nuanced understanding of how these features impact the overall output of the model. By leveraging SHAP, we enhance interpretability and transparency in the complex landscape of machine learning, empowering practitioners to gain valuable insights into the factors driving model predictions.

### Statistical analysis

All statistical analyses were executed using R software (version 4.2.1, https://www.r-project.org/). The conducted statistical tests were two-tailed, and significance was determined with a threshold of *P* < 0.05. The model development process incorporated various R packages, namely “tidyverse,” “survival,” “mlr3verse,” “mlr3proba,” “mlr3extralearners,” and “survex.” Additionally, the construction of the web-based dynamic model was facilitated by the “shinydashboard” R package. This comprehensive utilization of R packages signifies the incorporation of diverse statistical and machine learning tools, ensuring a robust and multifaceted approach in the development and analysis of the model.

## Results

### Patient characteristics

Out of the initial cohort of 2,706 patients diagnosed with ASC who were eligible for consideration, 241 individuals met the predefined inclusion criteria and were included in the study. The median survival duration was found to be 35 months, with a significant 73.03% of the total cohort succumbing to mortality. In terms of demographics, the majority of the cohort comprised individuals of Caucasian ethnicity (83.82%) and those aged 65 years or older (71.78%).

The primary tumor was most commonly located in the upper lobes of the lungs, accounting for over 68% of cases, followed by occurrences in the lower lobes at 24.90%. Tumor sizes less than 3 cm were more prevalent, observed in 42.32% of patients compared to other size subgroups. Regarding perioperative interventions, over 65% of patients underwent lobectomy resection, 12.45% underwent wedge/segmental resection, 7.88% underwent bilobectomy, and 13.69% underwent pneumonectomy. Only four patients (1.66%) received surgery in other anatomical regions/sites. Positive regional nodes were identified in over 80% of the patients, of whom 56.85% had 1to 3 nodes positive, and 25.31% had more than 3 nodes positive. Radiotherapy was administered to approximately 40% of individuals during the perioperative period, and systemic therapy was employed in over 67% of the surgical population, with over 53% of them receiving therapy after surgery. Throughout the course of oncological interventions, approximately 40% of patients received radiotherapy, while 67% underwent chemotherapy. A comprehensive summary of baseline characteristics is provided in Table 1.

**Table 1:** Baseline characteristics of stage III ASC patients post-tumor resection.

Features	Total (*n* = 241)
Age (years), *n* (%)	
<65	68 (28.22)
≥65	173 (71.78)
Sex, *n* (%)	
Male	139 (57.68)
Female	102 (42.32)
Race, *n* (%)	
White	202 (83.82)
Black	18 (7.47)
Others	21 (8.71)
Marital Status, *n* (%)	
Married	140 (58.09)
Unmarried	92 (38.17)
Unknown	9 (3.73)
Primary tumor site, *n* (%)	
Upper lobe	165 (68.46)
Middle lobe	9 (3.73)
Lower lobe	60 (24.90)
Overlapped lesions	7 (2.90)
Tumor size (cm), *n* (%)	
0–3	102 (42.32)
3.1–5	74 (30.71)
5.1–7	30 (12.45)
>7.1	35 (14.52)
Tumor grade, *n* (%)	
Grade I	2 (0.83)
Grade II	78 (32.37)
Grade III	147 (61.00)
Grade IV	2 (0.83)
Unknown	12 (4.98)
Tumor laterality, *n* (%)	
Left	105 (43.57)
Right	136 (56.43)
T stage, *n* (%)	
T1	35 (14.52)
T2	82 (34.02)
T3	78 (32.37)
T4	46 (19.09)
N stage, *n* (%)	
N0	23 (9.54)
N1	59 (24.48)
N2	152 (63.07)
N3	7 (2.90)
AJCC stage	
IIIA	222 (92.12)
IIIB	19 (7.88)
Surgery of primary tumor site, *n* (%)	
Wedge/segmental resection	30 (12.45)
Lobectomy	159 (65.98)
Bilobectomy	19 (7.88)
Pneumonectomy	33 (13.69)
Surgery of other region/sites, *n* (%)	
Yes	4 (1.66)
No	237 (98.34)
Regional nodes examination, *n* (%)	
Negative	35 (14.52)
1–3 node(s) positive	137 (56.85)
>3 nodes positive	61 (25.31)
Not examined	8 (3.32)
Radiation sequence with surgery, *n* (%)	
No radiation	145 (60.17)
Radiation after surgery	87 (36.10)
Radiation prior to surgery	9 (3.73)
Systemic therapy and surgical procedures, *n* (%)	
No systemic therapy	79 (32.78)
Systemic therapy after surgery	130 (53.94)
Systemic therapy before surgery	18 (7.47)
Systemic therapy both before and after surgery	14 (5.81)
Radiotherapy, *n* (%)	
Yes	96 (39.83)
None/unknown	145 (60.17)
Chemotherapy, *n* (%)	
Yes	162 (67.22)
None/unknown	79 (32.78)
Status, *n* (%)	
Alive	65 (26.97)
Dead	176 (73.03)
Survival time(days), Mean ± SD	35.33 ± 28.6

SD, standard deviation.

### Identification of prognostic features and model development

**Figure 1:**
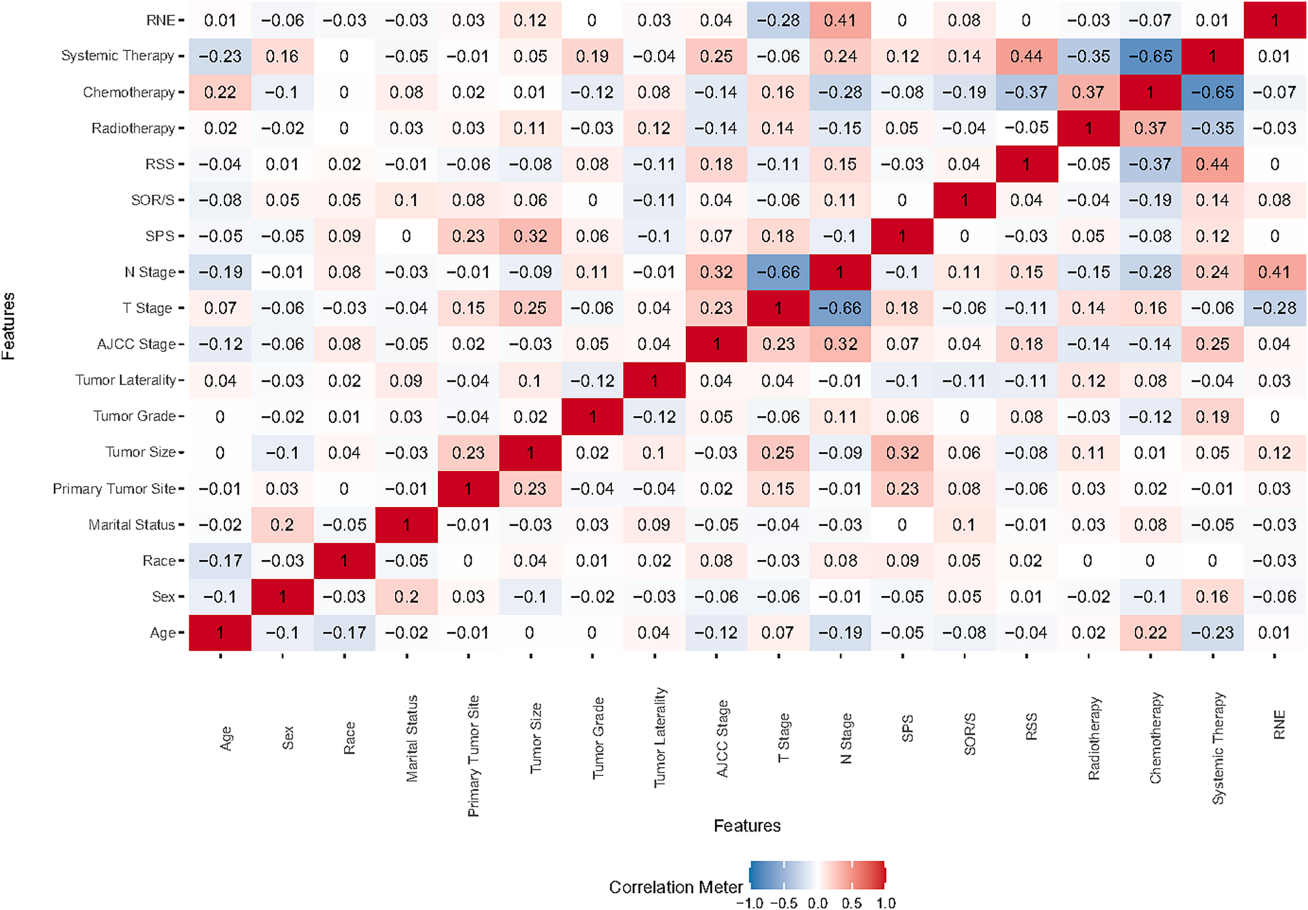
Heat map illustrating feature correlations. SOR/S, surgery of other region/sites; RSS, radiation sequence with surgery; SPS, surgery of primary site; RNE, regional nodes examination.

**Figure 2:**
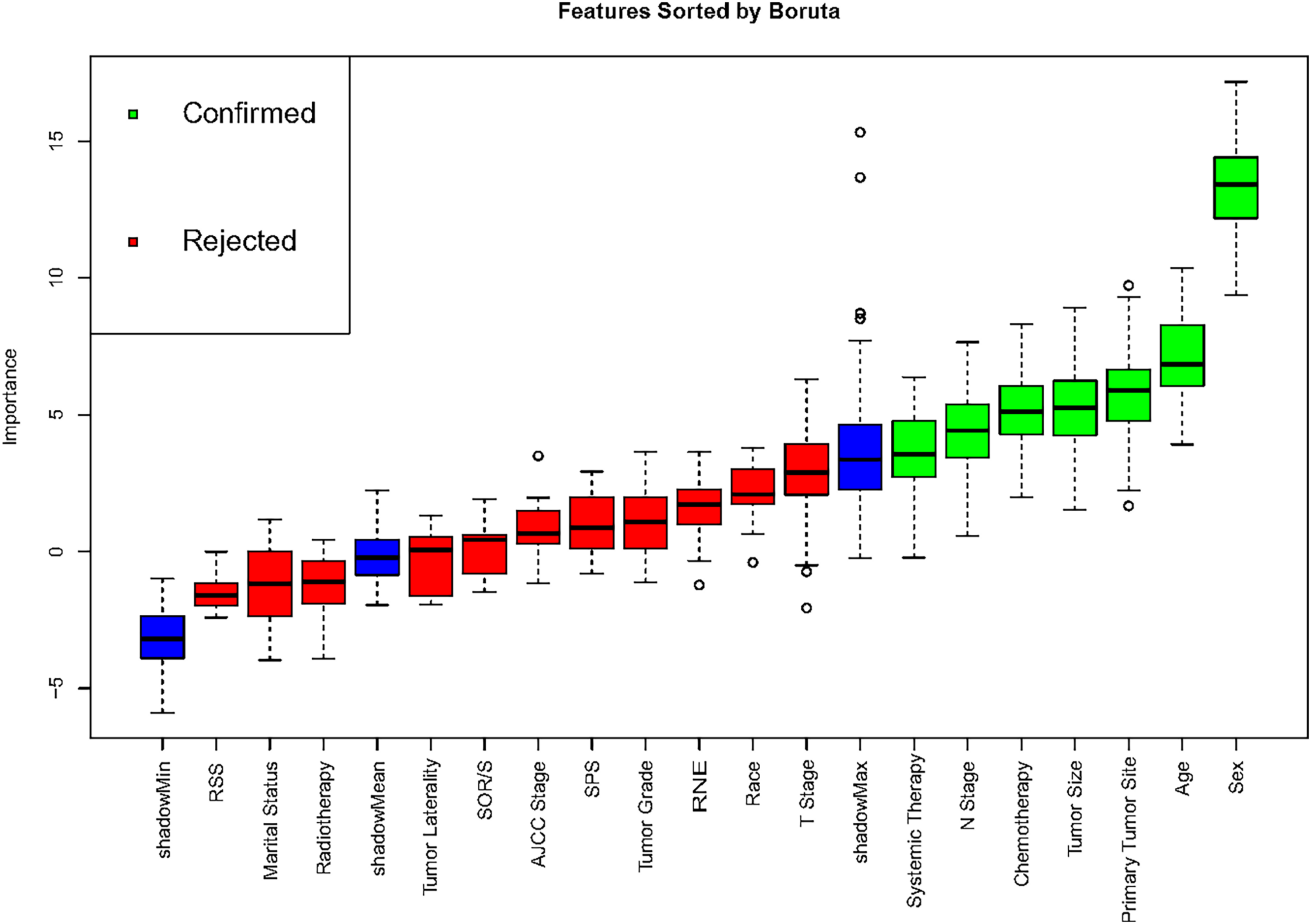
Prognostic features sorted by the boruta analysis. SOR/S, surgery of other region/sites; RSS, radiation sequence with surgery; SPS, surgery of primary site; RNE, regional nodes examination.

Correlation analysis is commonly employed to examine the interrelationships among data features. To ascertain the independence of these features, we conducted a Spearman correlation analysis and generated a correlation heat map. As depicted in Figure 1, this visual representation clearly shows a lack of significant correlation among the 18 features under investigation. We then performed a comprehensive examination of various features—including demographic characteristics, tumor attributes, and treatment methods—using them as covariates in a Boruta analysis. This analysis identified seven significant determinants of survival for ASC patients: age, sex, primary tumor size, N stage, chemotherapy, and systemic therapy. The outcomes of this analysis are detailed in Figure 2.

Building upon these findings, we developed a traditional prognostic model based on Cox regression. Furthermore, we enhanced our approach by constructing three additional prognostic models using machine learning techniques, aiming to improve the accuracy of patient survival predictions.

### Discriminatory ability of the predictive models

**Figure 3:**
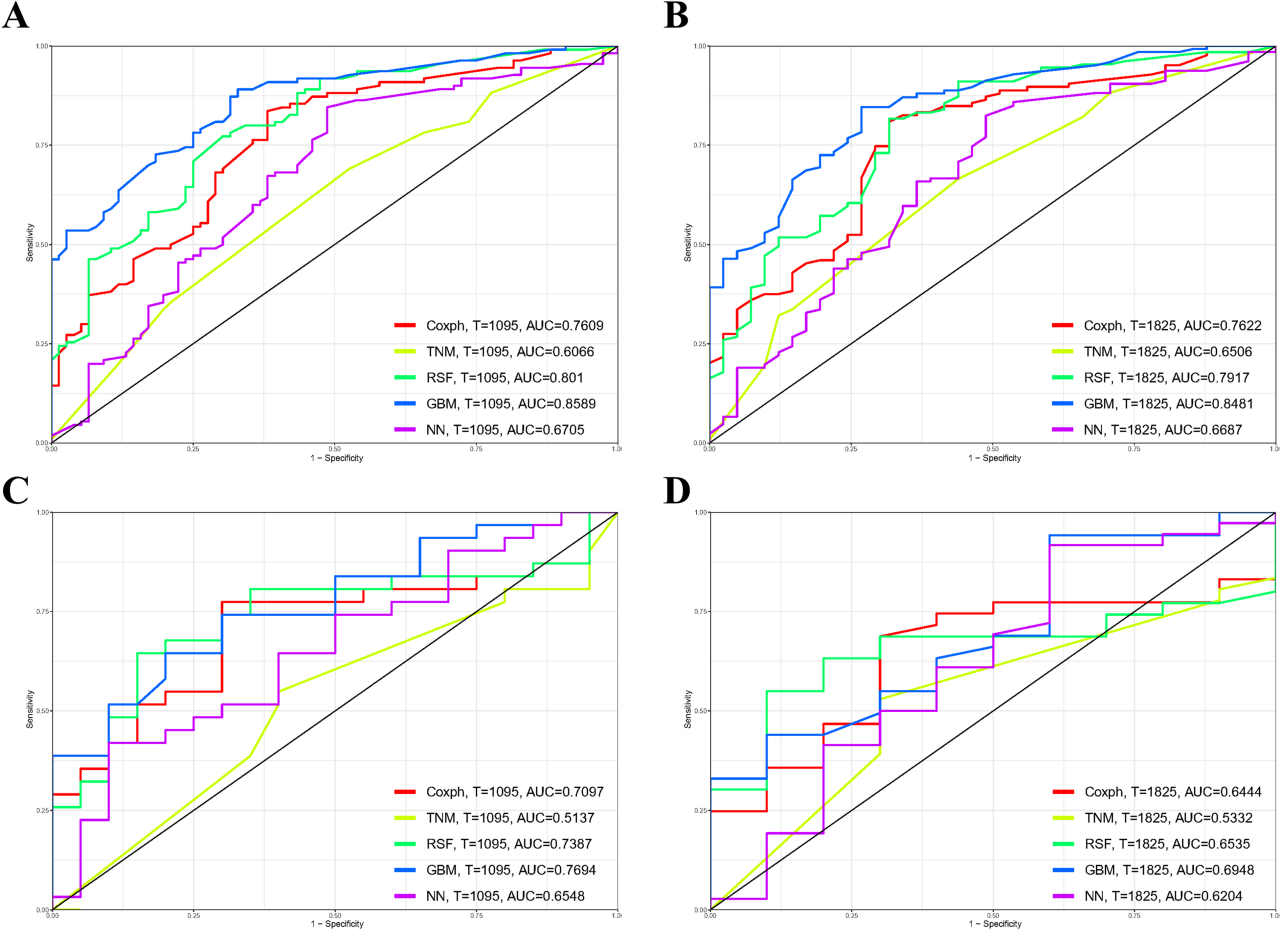
Comparison of prognostic models for 3- and 5-year overall survival (OS) prediction across various models: receiver operating characteristic curves illustrating the prediction of 3-year **(A**,**C)** and 5-year **(B**,**D)** OS in the training cohort **(A**,**B)** and validation cohort **(C**,**D)**. GBM, gradient boosting model; RSF, random forest; NN, neural network.

Referring to Figure 3, the results from the training cohort highlight the robust discriminatory capabilities of the Cox methodology, with notable AUC values of 0.761 (95% CI 0.692–0.83) and 0.762 (95% CI 0.678–0.846) for 3- and 5-year OS, respectively. However, in the validation cohort, the model showed lower AUCs of 0.71 (95% CI 0.566–0.853) and 0.644 (95% CI 0.473–0.816) for 3- and 5-year OS, respectively. In contrast, the TNM staging methodology exhibited comparatively lower discriminative power, yielding AUCs of 0.607 (95% CI 0.526–0.687) for 3-year and 0.651 (95% CI 0.557–0.745) for 5-year OS in the training cohort. These trends persisted in the validation cohort, where AUCs for 3-year and 5-year OS were 0.514 (95% CI 0.355–0.673) and 0.533 (95% CI 0.355–0.711), respectively.

**Figure 4:**
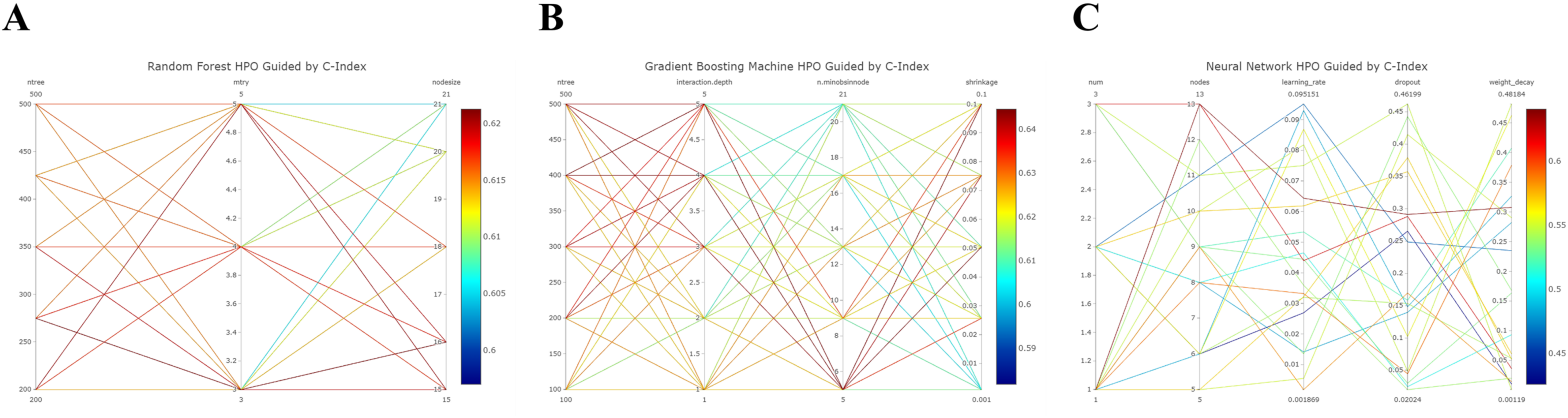
Refined hyperparameter optimization (HPO) process for three distinct machine learning models: random forest **(A)**, gradient boosting **(B)**, and neural network **(C)**.

The Random Forest model demonstrated commendable performance in both the training and validation cohorts, achieving AUCs of 0.801 (95% CI 0.738–0.865) and 0.792 (95% CI 0.712–0.872) for 3- and 5-year OS in the former, and 0.739 (95% CI 0.599–0.879) and 0.654 (95% CI 0.496–0.811) in the latter. The Gradient Boosting model displayed good discrimination, producing AUCs of 0.859 (95% CI 0.807–0.911) and 0.848 (95% CI 0.785–0.911) for 3- and 5-year OS in the training cohort, and 0.769 (95% CI 0.642–0.896) and 0.695 (95% CI 0.521–0.869) in the validation cohort. Lastly, the Neural Network model demonstrated lower discriminatory power with AUCs of 0.671 (95% CI 0.59–0.751) and 0.669 (95% CI 0.57–0.767) for 3- and 5-year OS in the training cohort, and 0.655 (95% CI 0.502–0.808) and 0.620 (95% CI 0.406–0.834) in the validation cohort. Figure 4 illustrates the process of hyperparameter optimization during the training of the three machine learning models.

### Assessment on calibration, brier score, and DCA

**Figure 5:**
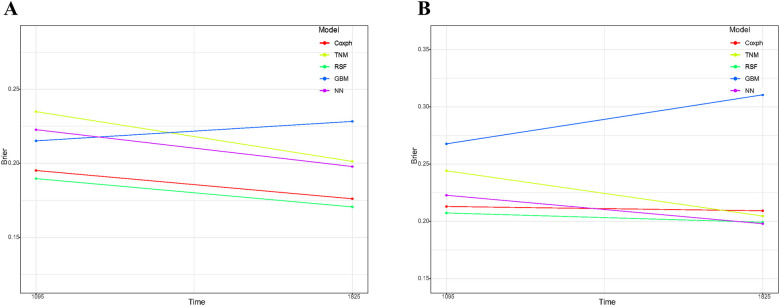
Comparative analysis of brier scores for 3- and 5-year overall survival prediction across various models in training cohort **(A)** and validation cohort **(B)** GBM, gradient boosting model; RSF, random forest; NN, neural network.

**Figure 6:**
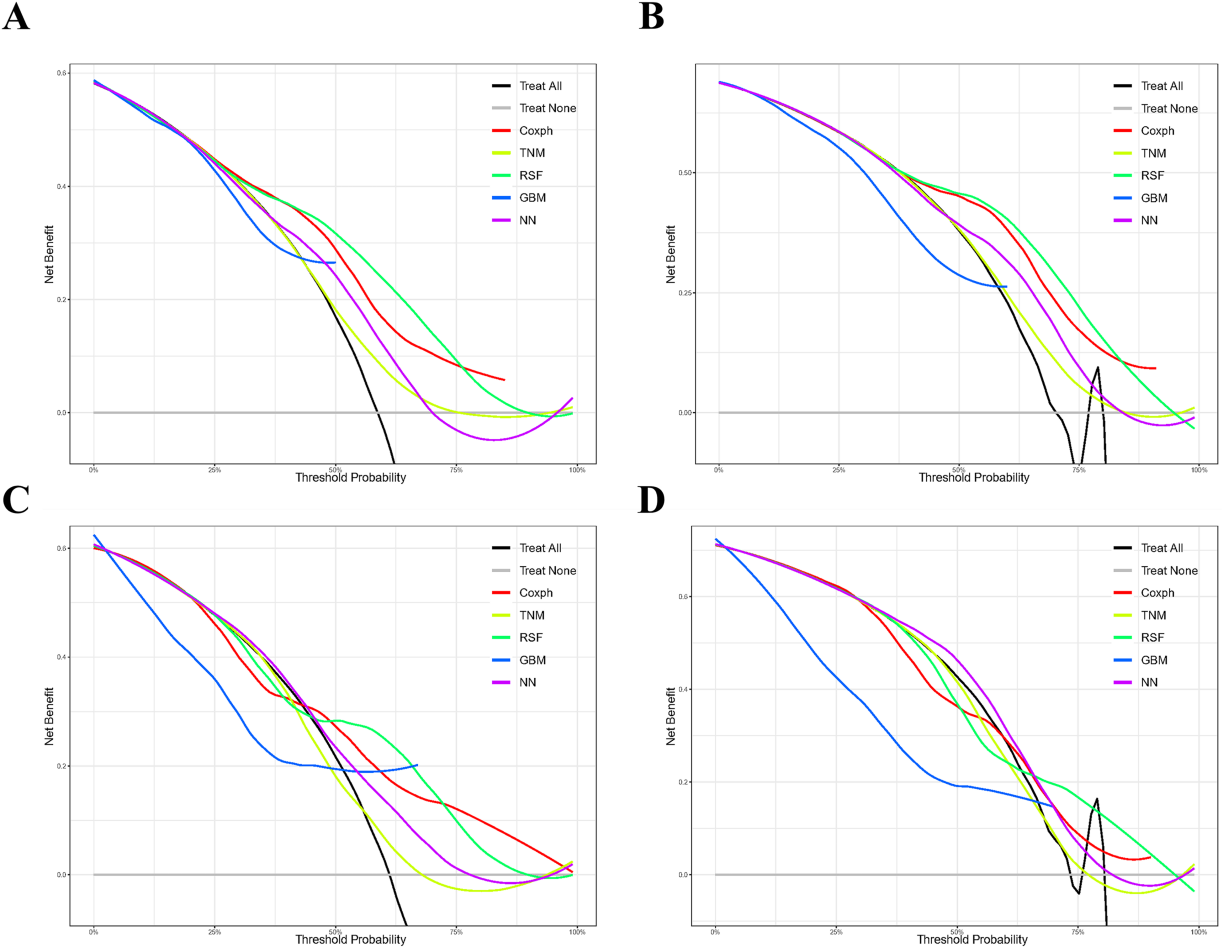
Comparison of prognostic models for 3- and 5-year clinical utility. Decision curve analysis illustrating the 3-year **(A**,**C)** and 5-year **(B**,**D)** outcomes in the training **(A**,**B)** and validation **(C**,**D)** cohorts. Models compared include Gradient Boosting (GBM), Random Forest (RSF), and Neural Network (NN).

As depicted in Supplementary Figure S1, the calibration plots reveal a remarkable alignment between the predicted and observed 3- and 5-year survival rates across both the Random Forest and Cox models. Notably, while the gradient boosting model achieved satisfactory AUC values, it exhibited the least favorable performance in calibration. The Brier score analysis detailed in Table 2 underscores the exceptional performance of the Random Forest model in both the training and validation cohorts. It registered indices of 0.189/0.171 (training) and 0.207/0.199 (validation) for 3- and 5-year OS predictions, respectively, outclassing its counterparts. It is worth noting the comparative performance of the Gradient Boosting model, which exhibited the least favorable outcomes, followed by the TNM staging and Neural Network models. Figure 5 provides a visual depiction of the Brier scores across the models. Figure 6 further illustrates the significantly positive outcome derived from the Random Forest model's assessment of mortality risk in the DCA, surpassing both traditional and machine learning models within the datasets. Cumulatively, these observations underscore the substantial clinical relevance of the Random Forest model in prognosticating the OS of stage III ASC patients post-tumor resection.

**Table 2:** Comparison of the brier score among the models.

Model	Times	Brier score	SE	95%CI
Random forest				
Training cohort				
	3-year	0.189	0.009	0.171–0.208
	5-year	0.171	0.013	0.144–0.197
Validation cohort				
	3-year	0.207	0.019	0.170–0.244
	5-year	0.199	0.026	0.148–0.250
Gradient boosting				
Training cohort				
	3-year	0.215	0.018	0.180–0.250
	5-year	0.228	0.017	0.194–0.262
Validation cohort				
	3-year	0.268	0.043	0.183–0.352
	5-year	0.310	0.044	0.225–0.396
Neural network				
Training cohort				
	3-year	0.224	0.009	0.205–0.243
	5-year	0.197	0.014	0.170–0.227
Validation cohort				
	3-year	0.223	0.018	0.188–0.257
	5-year	0.198	0.028	0.142–0.254
Coxph				
Training cohort				
	3-year	0.195	0.012	0.171–0.219
	5-year	0.176	0.015	0.146–0.206
Validation cohort				
	3-year	0.213	0.024	0.165–0.260
	5-year	0.209	0.029	0.15–0.268
TNM staging				
Training cohort				
	3-year	0.235	0.009	0.218–0.252
	5-year	0.201	0.013	0.175–0.227
Validation cohort				
	3-year	0.244	0.022	0.200–0.288
	5-year	0.205	0.031	0.144–0.265

SE, standard error; CI, confidence interval.

### Model interpretation

**Figure 7:**
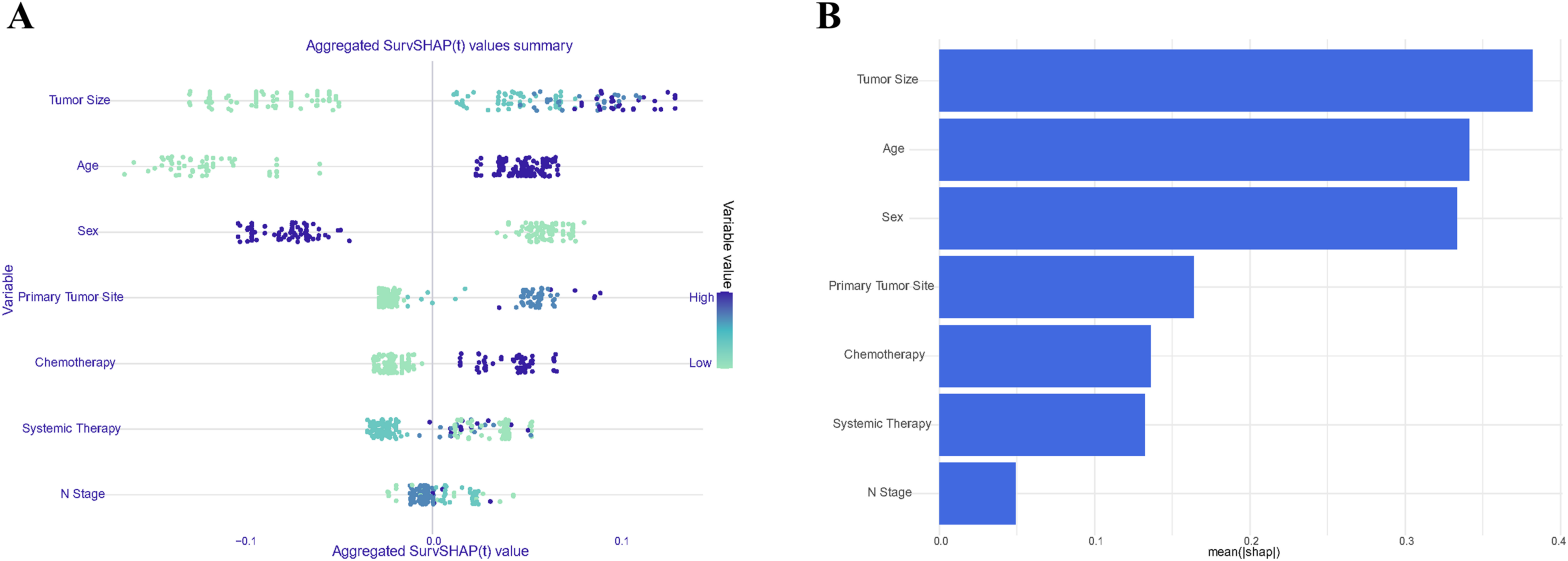
Summary plots of SHAP values in the random forest model. Sorted by sum of SHAP values across all patients **(A)**; Standard bar chart sorted by average absolute Shapley value **(B)**.

Given the remarkable efficacy demonstrated by Random Forest model in predicting OS within the datasets, we employed SHAP (SHapley Additive exPlanations) plots to elucidate the hierarchical significance of features and understand their respective impacts on prognosis within the established model. In Figure 7A, a discernible pattern emerges, indicating that features with higher SHAP values correspond to an increased probability of poor prognosis in stage III ASC patients post-tumor resection. The color spectrum in the plot provides additional insight, with green denoting small eigenvalues, light blue signifying eigenvalues close to the mean, and dark blue indicating large eigenvalues. Illustratively, the predominant feature in the figure emphasizes that tumor size has a notable impact on mortality incidence, followed by age, sex, and the primary tumor site. Figure 7B displays the mean SHAP of the selected features, further reinforcing the pivotal role of features in patients’ OS.

**Figure 8:**
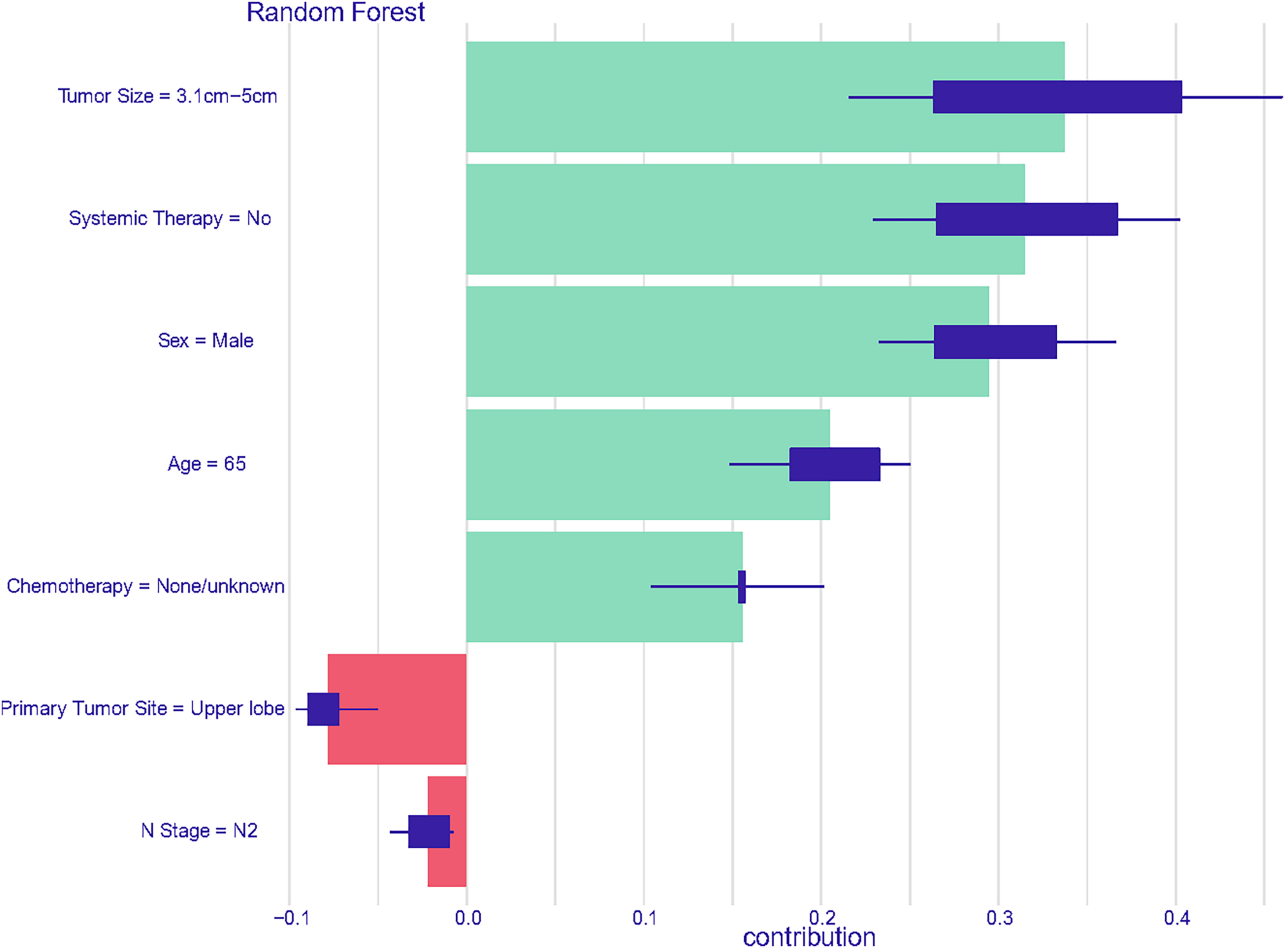
Prediction analysis utilizing features derived from the first member of the cohort in the random forest model.

Consequently, a unique individual was purposefully selected from the group to examine how various features would influence the outcome for the patient. As depicted in Figure 8, factors such as larger tumor size, absence of systemic therapy during the perioperative period, male gender, advanced age, and lack of chemotherapy throughout the disease course were identified as negatively impacting survival rates. Conversely, tumor growth in the upper lobe and the N2 stage were found to have a positive effect on the patient's prognosis. This comprehensive analysis is intended to emphasize and elucidate the key features that contribute to prognostic outcomes within the population under study.

### Development of a predictive system on a web server

Utilizing the Random Forest model as its foundation, a web-based tool has been created to streamline the predictive analysis of survival probabilities based on patient characteristics provided as input. This tool is designed to assist researchers who may not have expertise in machine learning, incorporating an automated methodology to configure, train, and evaluate the Random Forest model through a user-friendly interface (see Supplementary Figure S2). The model can be accessed through the following link: https://lungcare-innovators.shinyapps.io/ResectedTumorSurvival-LASC/.

## Discussion

In this comprehensive investigation, we conducted a meticulous analysis utilizing a cohort of 241 patients diagnosed with ASC who underwent primary tumor resection. Our study reveals that the Random Forest model surpasses traditional statistical methods like Cox regression and TNM staging, as well as machine learning models like Gradient Boosting and Neural Network, in its ability to predict OS among ASC patients. The beeswarm summary plot for the Random Forest model unveils tumor size as the most influential risk factor, followed by age, sex, and the primary site tumor growth. This insightful information enhances our understanding of prognostic factors in this subset population. Notably, we have developed a web-based individual prognostic tool based on the optimized Random Forest model, which holds promising implications for integration into clinical practice by providing clinicians with valuable insights for personalized patient care. Importantly, this study represents a pioneering exploration, marking the first instance in the existing literature of utilizing a machine learning-based prognostic model for ASC patients following primary tumor resection.

ASC poses a distinctive diagnostic challenge in contrast to ADC and SCC, given its composite nature that incorporates features of both entities. Inadequate sampling increases the risk of misclassification, leading to an erroneous categorization as either SCC or ADC (6). Existing literature has indicated that ASC exhibits a relatively aggressive clinical course and portends poorer survival outcomes compared to both ADC and SCC (3, 13). Therefore, a prompt and early intervention is important for effective management in this population. For surgical management in ASC, research conducted in two French centers reveals that patients undergoing surgical resection experienced a median OS of only 26 months, notably lower than the 46 months observed for ADC and 45 months for SCC (14). A comparable outcome was also noted in a single-center study in Japan, where ASC cases exhibited the least favorable survival following surgery. The 5-year survival rates for all-stage cases were 23.3% for ASC, 58.0% for ADC, and 40.8% for SCC (13). Similarly, a retrospective study conducted in Poland also confirmed that the outcome of surgically treated ASC patients was poorer than that of ADC, with a median OS of 20 months compared to 28.5 months in the latter (15). Regrettably, despite these studies confirming the poorer prognosis of ASC compared to ADC or SCC among patients undergoing surgical management, none of the studies delved into more refined subgroup analyses as well as developing prognostic models tailored to the population. Due to the rarity of ASC cases and the lack of clear management guidelines, particularly regarding the optimal timing of surgery for stage III NSCLC, there is a paucity of reports assessing the prognosis of stage III ASC patients who have undergone primary tumor resection. The management of stage III NSCLC is a subject of considerable debate within the medical community, primarily concerning the sequencing of surgery with chemotherapy and radiotherapy. This controversy arises because stage III NSCLC is a heterogeneous disease characterized by locally advanced tumors that may involve mediastinal lymph nodes (N2 or N3 disease), making treatment decisions complex and multifaceted. Consequently, it is of paramount importance to delineate the determinants influencing the prognosis of individuals and to construct robust prognostic models tailored to this specific patient subset.

The TNM staging system, a prevalent tool in cancer staging within clinical practice, encounters notable limitations that impact its prognostic efficacy. Firstly, it primarily relies on anatomical factors such as tumor size, lymph node involvement, and metastasis, neglecting important biological and molecular characteristics of tumors. This oversimplification fails to capture the heterogeneity within tumors, leading to inaccuracies in prognosis prediction. Secondly, TNM staging lacks dynamic assessment of cancer progression over time, as it is determined at a single point in time based on initial diagnostic findings. Consequently, it may not reflect changes in tumor behavior or response to treatment (16). These limitations highlight the critical need to incorporate additional clinical approaches and relevant parameters into cancer prognosis to improve its accuracy within clinical settings. In the realm of cancer prognosis modeling, machine learning holds significant promise in revolutionizing oncology modeling by surpassing the limitations of traditional methods such as COX proportional hazards regression and TNM staging. Unlike COX regression, which relies on assumptions of proportional hazards and may struggle with capturing complex nonlinear relationships, machine learning techniques can handle large, heterogeneous datasets with diverse types of features, including genomic, imaging, and clinical data. Machine learning models can uncover intricate patterns and interactions within these datasets, offering superior predictive accuracy and the potential for more personalized prognostic assessments. Moreover, machine learning approaches excel in integrating multiple sources of information, including molecular and biological markers, beyond the anatomical factors considered in TNM staging. This holistic approach enables a deeper understanding of tumor biology and behavior, leading to more precise prognostication and treatment recommendations (17–19). However, it's important to note that there's currently a lack of prognostic models tailored specifically for predicting survival outcomes in stage III ASC patients who have undergone primary tumor resection, whether utilizing advanced machine learning techniques or conventional algorithms.

Currently, several prognostic models are available to assess mortality risk integration and prognostication in individuals with ASC. For example, Liang et al. developed a nomogram specifically designed to prognosticate OS in ASC patients, leveraging a dataset of 4,600 patient records extracted from the SEER database. Their study identified nine key clinical factors influencing patient prognosis. The resulting model demonstrated a C-statistic of 0.755 in the training cohort and 0.721 in the validation cohort (20). Similarly, Wu et al. found that factors such as elderly age, male gender, absence of surgery, and advanced TNM stages independently predicted both OS and cancer-specific survival in ASC. Their model achieved a C-index of 0.79 for predicting OS probabilities in the cohort (21). Despite the robustness of these studies, the determinants associated with OS were found to be more broadly applicable to the general ASC population rather than offering precise insights tailored to specific substage ASC patients who have undergone particular interventions. This limitation likely stems from the varied determinants influencing OS within specific subgroups of lung cancer, such as tumor stage, degree of tumor differentiation, and responsiveness to treatment. In contrast, our model is uniquely designed to address the distinct characteristics of stage III ASC patients who have undergone surgery. This tailored approach enhances the precision of survival estimation within this cohort. Moreover, our model benefits from broad geographical coverage within the database and demonstrates commendable performance in machine learning prediction, further enhancing its prospective applicability to a wider population.

Regarding the prognostic assessment of patients with ASC, the Boruta algorithm identified age, sex, tumor size, primary tumor site, N stage, chemotherapy, and systemic therapy as independent prognostic features. These findings are consistent with findings from certain prior studies (20–23). Furthermore, some studies revealed that the histological subtype of ADC in ASC, visceral pleura involvement, and EGFR mutation had an influence on patients’ survival (15, 24, 25). However, due to the absence of such parameters in the database, our ability to delve into the nuanced impacts of these parameters on cancer prognosis is further hindered. It is crucial to acknowledge that the absence of such pivotal information may impact the predictive efficacy of the model.

To thoroughly evaluate the effectiveness of our model, we employed a robust 5-fold cross-validation approach to address concerns regarding overfitting and to validate its ability to generalize across diverse population subsets. The calibration curves, which illustrate the agreement between predicted and actual survival probabilities, serve as compelling evidence of the reliability of our Random Forest model. This confidence is further bolstered by the observation that the Brier Score demonstrated more favorable performance compared to alternative models considered. To reinforce the clinical relevance of our model, we conducted a comprehensive assessment using DCA curves to explore potential clinical implications. When compared to conventional models and two contemporaneously developed machine learning models, the Random Forest model exhibited superior performance, yielding higher net benefits in the validation cohort. This outcome highlights its considerable potential for implementation in clinical practice, suggesting it could significantly enhance decision-making processes and ultimately improve patient outcomes.

While our study successfully developed a machine learning model with commendable predictive efficacy for a specific patient subset, it is important to acknowledge several inherent limitations affecting various aspects of our investigation, including study design, data acquisition, model validation, and interpretation. Firstly, the retrospective nature of our study introduces potential selection bias due to reliance on historical data. Secondly, despite the extensive scope of the SEER database, it lacks detailed information on radiation regimens, dosages, specific chemotherapy and systemic therapy agents, and essential patient factors such as genetic mutations, blood test results, and comorbidities. Additionally, although we employed 5-fold cross-validation to mitigate overfitting, external validation is necessary to ensure the generalizability of our model beyond the current dataset. Recognizing these limitations is crucial for judicious interpretation of our findings and underscores the need for caution and further research to refine and validate our model for broader clinical applicability.

## Conclusions

This study marks the first instance of employing machine learning models to evaluate prognosis in patients who have undergone tumor resection for stage III ASC. By introducing this innovative personalized predictive tool, clinicians gain the capability to design treatment protocols precisely customized to the unique characteristics of individuals within this patient cohort. Furthermore, this tool facilitates the development of optimal follow-up schedules, thereby enhancing the efficacy and individualization of patient care strategies.

## Data Availability

Publicly available datasets were analyzed in this study. This data can be found here: https://seer.cancer.gov/data/.

## Supplementary material

Supplementary Figure S1 Calibration plots depicting the comparison of 3- and 5-year survival predictions among the constructed models, including Gradient Boosting (GBM), Random Forest (RSF), and Neural Network (NN).

Supplementary Figure S2 Online web server interface for the Random Forest model.
