# Supplementary figures and images for: Integrative prognostic modeling for stage III lung adenosquamous carcinoma post-tumor resection: machine learning insights and web-based implementation
Source: Front Surg. 2024 Oct 22;11:1489040. doi: 10.3389/fsurg.2024.1489040 (PMC11538581; doi:10.3389/fsurg.2024.1489040)

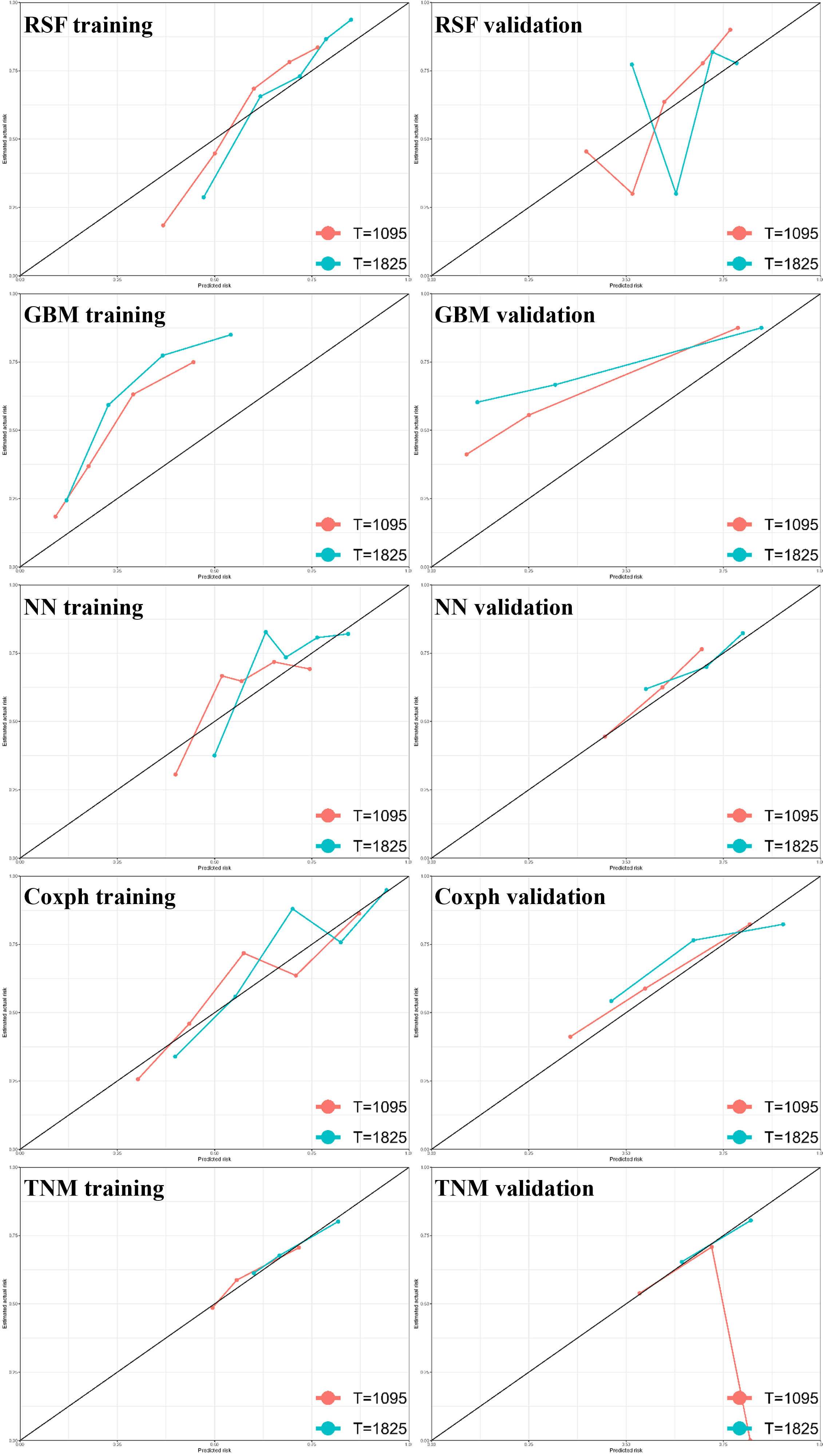

Supplement: Supplementary Figure S1 — Calibration plots depicting the comparison of 3- and 5-year survival predictions among the constructed models, including Gradient Boosting (GBM), Random Forest (RSF), and Neural Network (NN). [file Image1.png]

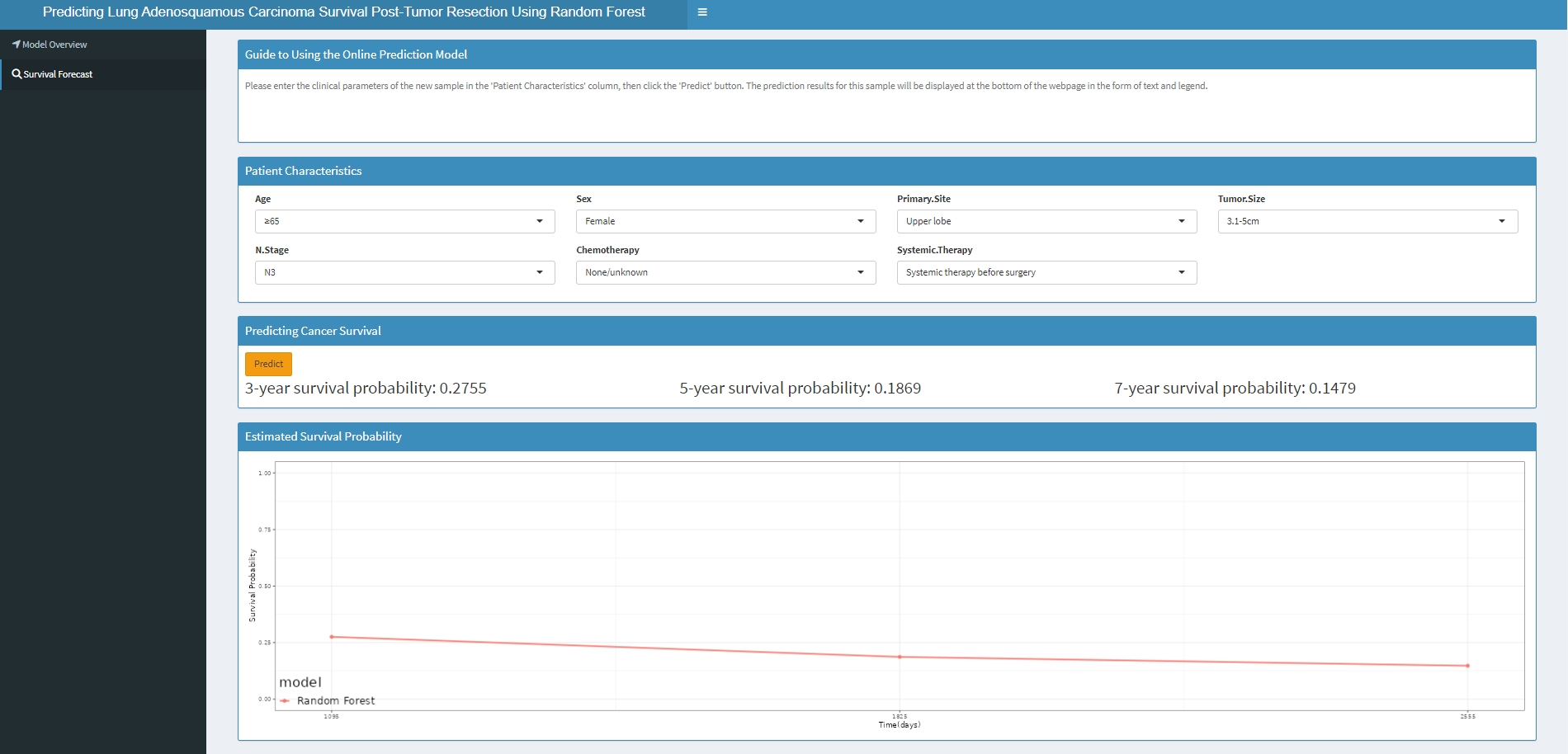

Supplement: Supplementary Figure S2 — Online web server interface for the Random Forest model. [file Image2.png]
